# Sonar Dome Geometry Design Using CFD to Reduce Ship Resistance at Cruise Speed

**DOI:** 10.3390/s22145342

**Published:** 2022-07-18

**Authors:** Ping-Chen Wu, Jiun-Yu Chen, Xuan-Hong Liu, Chen-I Wu, Chien-Chung Lu

**Affiliations:** 1 Department of Systems and Naval Mechatronic Engineering, National Cheng Kung University, Tainan City 70101, Taiwan; f14046038@gs.ncku.edu.tw (J.-Y.C.); p16104043@gs.ncku.edu.tw (X.-H.L.); p18051042@gs.ncku.edu.tw (C.-I.W.); 2 Fishing Boat and Marine Engineering Research Center, National Cheng Kung University, Tainan City 70101, Taiwan; em61550@email.ncku.edu.tw

**Keywords:** computational fluid dynamics (CFD), sonar dome, ship resistance, viscous flow, hull form optimization

## Abstract

The objective of this study is to design the hull-mounted sonar dome of a ship. The goal is to reduce the ship total resistance and improve the flow field around the sonar dome for the ship design speed. OpenFOAM 6 was applied to analyze the viscous flow around the ship bow and then optimize the sonar dome geometry. The length, width and depth of the original geometry were maintained. Only the local geometry was fine-tuned considering the back slope and front tip by using Rhinoceros 6. The verification and validation was performed for the original hull form against towing tank resistance data. The grid independence was checked for the optimal design in different design stages. To ensure less influence on the interior equipment installation and to be able to re-use the non-steel dome part, the best resistance reduction is almost 2%. With a larger allowance of shape deformation, the maximal reduction could reach slightly higher than 3%. The flow field is improved for smaller flow separation and vortex, and less fluid nose in sonar detection is expected. The main reason of the resistance reduction is the decrease of the pressure component. In conclusion, a sonar dome design procedure is proposed, and an optimal geometry is suggested.

## 1. Introduction

Relying on the rapid development and huge progress of computational technology, CFD (computational fluid dynamics) has become a useful tool for ship design. CFD and towing tank experiments can assist each other and make the best of their own advantages to achieve different design goals. By managing the simulation case size properly, CFD can be inexpensive and less time-consuming. Building a real ship model is not required for CFD study, and CFD is also not constrained by towing tank schedules and facility location. Moreover, the detailed analysis of local flow field is easier using CFD results because not every towing tank is capable of conducting PIV (particle image velocimetry) measurements. It is important for fluid dynamists to understand detailed flow phenomena and then make further modifications and improvements. Thus, ship engineers can utilize CFD in initial design stages. After analysis iteration between simulations and numerous different geometries, suitable or optimal ones or several optional designs can be chosen for further investigation, for example, towing tank experiments or more detailed CFD simulations.

The objective of the present work is to reduce the total resistance for a ship hull form integrated with a sonar dome at the ship bow. Based on the existing towing tank resistance data of the ship model, its hull-mounted sonar dome shape was further designed and optimized by using CFD. The design condition was cruise speed, i.e., the speed at which the ship advances most of the time. The resistance reduction would provide fuel savings, and the associated flow field improvements, such as smaller flow separation, could be beneficial to the sensor (sonar) detection capability. Therefore, the flow field phenomena, such as flow separation, boundary thickness and wake length around the sonar dome, were analyzed by CFD solution as well.

Regarding the hull form with sonar dome for academic research, DTMB (David Taylor Model Basin) 5415 is the most worthy one mentioned. Its geometry details and experimental data were released by the ONR (Office of Naval Research) in the early 1980s. Since then, it has been studied by many researchers. The design ship speed is at Fr (Froude number) equal to 0.25 and 0.41, corresponding to full scale 18 and 30 knots (for a 142 m long ship). On the other hand, CFD has been coupled by optimization algorithm or software to design the ship hull form of DTMB 5415 or its bow shape.

Kim et al. [1] considered nine parameters, including entrance angle, sonar dome height and size, etc. To change DTMB 5415 geometry, the shifting method based on sectional area curve and radial basis function interpolation were used. Ship resistance was estimated by Neumann–Michell linear theory, while seakeeping performace was evaluated by Bales’ ranking method. In conclusion, in Case-III at Fr = 0.28 and 0.45, the lowest resistance was achieved with the highest seakeeping rank. Compared with the original 5415 hull, the wet area and displacement only increased 1% and 1.6%, respectively, for the optimal hull form.

The software CAESES was utilized by Feruglio [2] to parameterize the DTMB 5415 hull. For the ship bow, the parameter of FFD (free-form deformation) considered the length, width, dpeth and angle of the bulbous bow. The ship stern shape was re-built by NURBS (nonuniform rational B-spline) curves. The ship resistance was predicted by using OpenFOAM. Two stages were performed to design the 5415 hull. First, 60 different hull geometries were analyzed, and then according to the result, the parameter range was narrowed down to select 40 geometry changes. Linear, Kriging and ANN (artificial neural network) were optional methods. Finally, they realized that the optimal solution had been achieved in the first stage, which reduced 9.94% resistance at Fr = 0.28. During optimization, it was found the resistance increases as the bow becomes wider and deeper, and a shorter bow decreases the resistance.

In Zhang et al. [3], Opt LHD (optimal Latin hypercube design) and NLPQL (non-linear programming by quadratic Lagrangian) were combined for optimization. The geometry change was performed by using ASD (arbitrary shape deformation) based on B-spline in the commerical software Sculptor. CFD software Star-CCM+ was used, in which the flow field and rigid body motion solver were coupled by overset grid and DFBI (dynamic fluid body interaction). The free surface and waves were modeled by VOF (volume of fluid). After optimization, the sonar dome of the model 5512 hull form (BTMB 5415’s geosim) protuded forward becoming longer and sharp. In calm water, the resistance per displacement reduced by 1.56, 3.04 and 3.89% for Fr = 0.19, 0.28 and 0.34, respectively. Instead, in waves (wave length equal to ship length) the difference of the heave and pitch motion response was not obvious. At one-quarter wave ecounter period (*T_e_*), the original and optimal 5512 hull had the shollowest draft. At three-quarters *T_e_*, both drafts reached their deepest. However, the lower pressure distribution on the optimal sonar dome appeared lower. Additionally, at one-quarter, one-half and three-quarters *T_e_*, the smaller ship-making wave amplitude around the ship bow and shoulder was found.

Tahara et al. [4] used CFDSHIP-IOWA for ship resistance simulation. Model 5415-A was optimized by MOGA (multi-objective genetic algorithm), and CAD (computer-aided design) for geometry changes. Model 5415-B was the result of the optimizer UNICO (uniform covering) with FFD. The seakeeping performance was evaluated by strip theory for both. At Fr = 0.28, the resistance reduction was 5.02% and 3.78% for 5415-A and -B, respectively. The seakeeping index function for Fr = 0.28 and 0.41 was reduced around 1% for 5415-A and 2% for 5415-B. In addition, the towing tank test was conducted for 5415-B and 4.75% resistance reduction was measured. Later, FLOWPACK was deveopled by Tahara et al. [5]. Using MOGA along with a CAD module named NAPA, the ship shape was modified by changing IGES (initial graphics exchange specification) format and outputed for the grid generator. First, by manually controlling NAPA, the ship resistance was lowered up to 10.1%. Next, the design constraints were exerted strictly on the automatically executed NAPA. However, due to slightly increased displacement, the resistance reduction was 7.8%. Compared with the original geometry, the maximal width of the optimal sonar dome decreased 7.7% but elongated 39.5% with a smaller entrance angle and curved tail. The conclusion supported the geometry change trend in [4], but a further improved result was provided.

Diez et al. [6] and Grigoropoulos et al. [7] designed the 5415 ship hull by using three optimization methods based on potential flow theory, INSEAN/UI, NTUA and ITU, and then used CFD software ISIS-CFD to evaluate the result. The design condition was Fr = 0.25 and 0.41. The design target was the resistance ratio F1, and seakeeping performance index F2 representing the vertical acceleration of s the hip bridge. F1 and F2 were both weighted and standardized. The most accurate method was NTUA, which under-estimated F1 by 6.1%. At Fr = 0.25, 8.8% resistance reduction was gained, but the resistance increased 3% at Fr = 0.41. F2 was merely over-predicted by 0.7%, and from the original 5415 to optimal solution F2 reduced by 6%. In INSEAN/UI, the resistance was calculated by integrating the pressure on the double body model with Neumann–Kelvin linearization. The ship motion solver was strip theory. The optimizer was MODPSO (multi-objective extension of the deterministic particle swarm optimization). The geometry was deformed by orthogonal function. ITU used the Dawson method to estimate resistance, and the strip theory program SHIPMO for ship motion. The optimization method combined neural networks and SQP (sequential quadratic programming). The shape was modified by Akima cubic B-spline. The resistance and ship motion program for NTUA were SWAN2 and SPP-86, respectively. The optimization was performed by NSGA-II (non-dominating sorting genetic algorithm-II). The geometry was changed by the commerical software CAESES.

In this study, the CFD solver is OpenFOAM 6 without consideration of ship motions. The design target is the ship total resistance, and the flow field is analyzed around the sonar dome at the ship cruise speed. The main particulars of the hull geometry were kept at the fixed length, width and depth of the original sonar dome. The control points of NURB surface on sonar dome are adjusted locally and manually to generate new geometry by using Rhinoceros 6. The main considerations for geometry changes are the dome back slope (trailing edge) and its front tip (leading edge).

## 2. Geometry and Test Conditions

**Figure 1:**
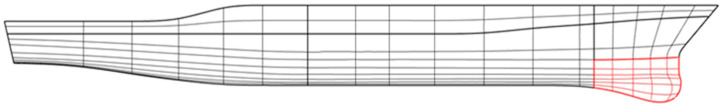
Ship model (**side view**). The red lines indicate the sonar dome.

The ship model length (length between perpendiculars) is *L* = *L_PP_* = 3 m. The beam (maximum beam of waterline) is *B_WL_* = 0.39 m and the draft is *t* = 0.115 m. The Block coefficient is *C_B_* = 0.52. The ship model was in bare-hull condition in this study, as shown in Figure 1. The calm water resistance experiment was conducted in the towing tank at National Cheng Kung University. The environmental conditions were at water temperature 26.2 °C, so the water density was *ρ* = 996.73 kg/m^3^ and the dynamic viscosity was *v* = 8.6905 × 10^−7^ m^2^/s. The ship model speed was 1.248 m/s, corresponding to Froude number Fr = 0.23 and Reynolds number *Re* = 4.307 × 10^6^. Fr = 0.23 is the cruise speed of the ship.

**Figure 2:**
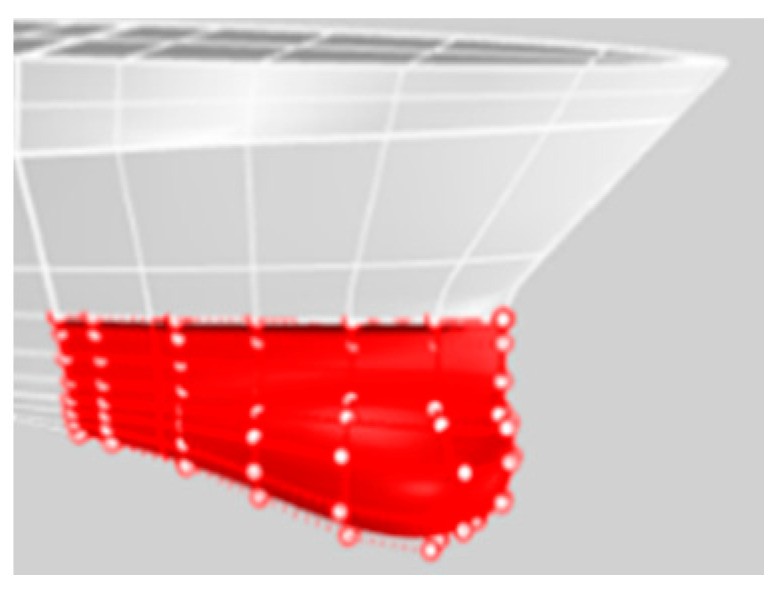
Control points on sonar dome surface.

In Figure 1, the focus of the present work, the sonar dome is marked by the red lines. Its surface geometry is modified for optimization, but the other part of the ship hull is not changed. The geometry change method is to adjust the control points of the NURBS surface to generate a sonar dome surface (see Figure 2).

**Figure 3:**
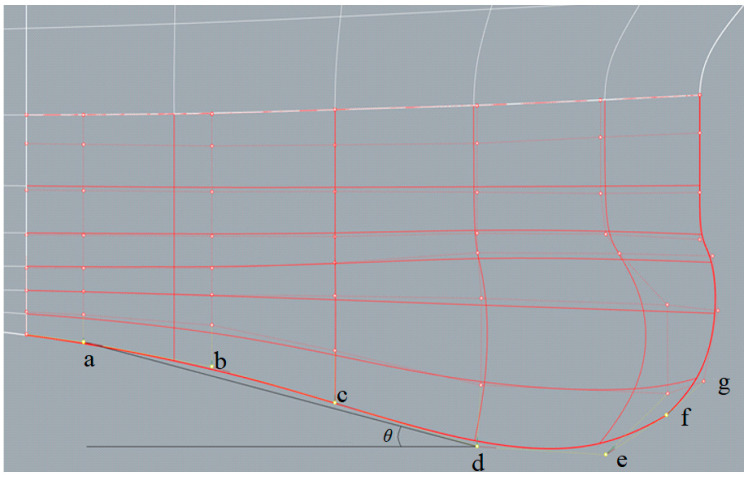
Control point a, b, c, d, e, f, g along the sonar dome bottom.

**Figure 4:**
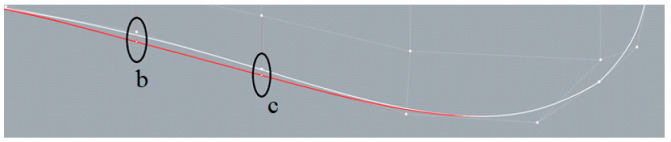
Control point b and control point c in Step (2).

Figure 3 indicates the major control points used here, which are mainly along the bottom of the sonar dome. The back slope of the sonar dome is the main target since its shape was found to be concave, e.g., control points (pt.) b and c are located inward, away from lines a–d. It was expected to cause negative influences, such as separation flow (explained in Section 4). The geometry change procedure is summarized as follows:

(1) Adjust the back slope *θ* as defined in Figure 3 between lines a–d and the horizontal axis originating from the control point d to downstream.
(2) Move pt. b and c to lines a–d. An example is demonstrated in Figure 4.
(3) Further improve the detailed geometry (e.g., front-edge fairing) for selected cases.

**Figure 5:**
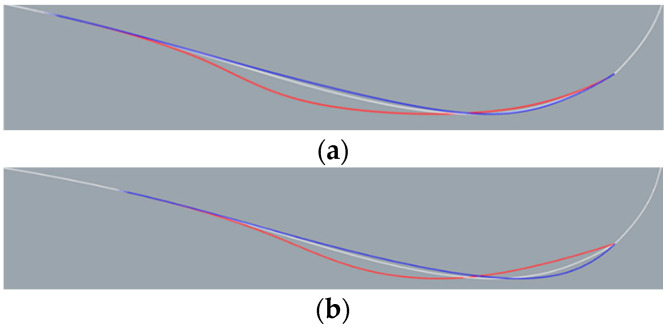
Step (1) result: *θ* = 14.113° (red line), 14.967 (white line), and 18.718° (blue line). (**a**) Type A and (**b**) Type B.

The sonar dome design splits into two types in step (1): Types A and B. Type A ensures less influence on the interior equipment installation and is able to re-use the non-steel dome part. Thus, only pt. d is shifted horizontally. Type B is designed to allow larger shape deformation. Therefore, pt. d and e were shifted together horizontally. Figure 5a,b shows the result of step (1) for Types A and B, respectively. For Type A, *θ* = 14.113° (red line)–18.718° (blue line). For Type B, *θ* = 14.113° (red line)–17.414° (blue line). The *θ* range was based on the trend of resistance result presented in Section 4, and *θ* = 14.113°, 17.414° and 18.718° corresponds to pt. d shifting 0.03 m upstream, 0.07 m downstream and 0.1 m downstream, respectively. The original sonar dome has *θ* = 14.967°, corresponding to the white line in Figure 5a,b for the same geometry.

**Figure 6:**
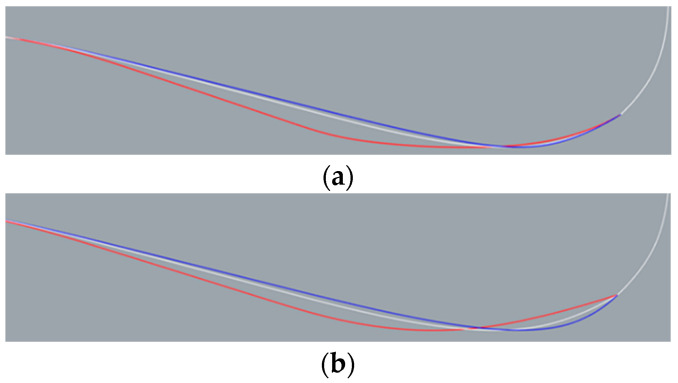
Step (2) result: *θ* = 14.113° (red line), 14.967 (white line), and 18.718° (blue line). (**a**) Type A and (**b**) Type B.

Figure 6 shows the result of step (2) for both types. For Type A, the major deformation is the downward inflation of the back slope. For Type B, the geometry change includes a modified back slope similar to Type A but with much more shrinking at the front bottom.

The nomenclature of the sonar dome configuration is A*θ* or B*θ*, e.g., *θ* = 14.967° corresponds to A14.967 and B14.967. They are the original sonar dome with the step (2) change, as shown by the white line in Figure 6a,b. Except for A14.967 = B14.967 (both are the same geometry), the Types A and B geometry are different for the other *θ*s.

**Figure 7:**
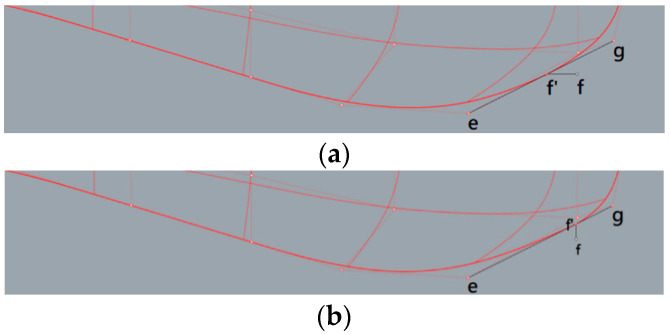
Front-edge fairing by moving control point f to control point f’. (**a**) B17.018h, (**b**) B17.018v. (Control point e and control point g are maintained in the original position).

In step (3), the optimal Type B, i.e., B17.018 (see Section 4.2), was selected to perform front-edge fairing to smooth the discontinuous and sharp tip caused by the much more significantly shrinking front bottom mentioned previously. Two kinds of front-edge fairing were performed as indicated in Figure 7: depending on moving pt. f to f’ horizontally or vertically, B17.018h or B17.018v, respectively, was generated.

## 3. CFD Methods

The CFD tool in the present work is OpenFOAM (Open Source Field and Manipulation) V6. The two-phase incompressible flow model VOF (volume of fluid [8]) was used to consider the free-surface effect and the ship’s wave-making resistance, which was included in the ship total resistance measured in the towing tank. The velocity and pressure field, turbulence and free surface elevation were solved around the ship’s SST (shear stress transport) *k-ω* turbulence model [9], and the PIMPLE velocity and pressure coupling algorithm [10] were utilized. The numerical schemes of the flow field solver are listed as follows: the first-order Euler implicit method with local time stepping for time differential term, the first-order central difference for gradient term, the second-order upwind method for the divergence term, the linear interpolation for Laplacian term and control point value between two volumes (or two surfaces) and the second-order explicit scheme with non-orthogonal correction for the surface normal direction gradient.

**Figure 8:**
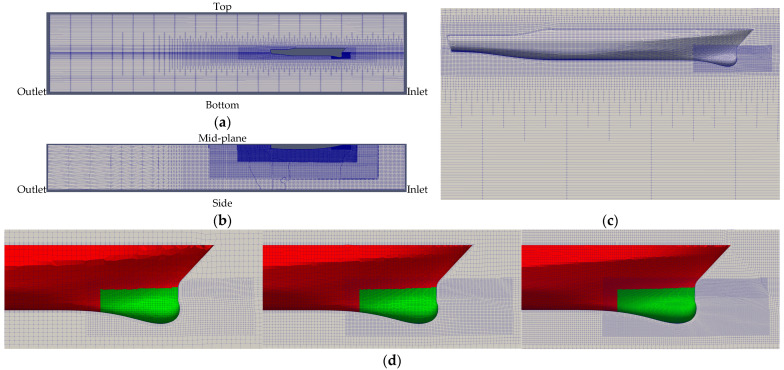
Grid topology. (**a**) Far-field grid distribution (**size view**), (**b**) far-field grid distribution (**top view**), (**c**) grid distribution around ship hull, (**d**) grid distribution around ship bow and sonar dome (**Left** to **right**: coarse, medium, fine grids).

Only half-sized flow field (star-board side) was simulated because of the flow symmetrical conditions. The computational domain lengths were around 3 *L* and 1 *L* before ship front and aft perpendicular, respectively, which were long enough to avoid truncation error. According to the towing tank size, the distances were 4 m to the bottom and 4 m to the side. Refer to Figure 8a,b for the domain size. The finer grid density can be seen allocated near the free surface and the ship body allocated gradually from the boundaries. Since the sonar dome was our design target, an additional grid refinement covering the ship bow under water was added to describe the geometry changes and details, as shown in Figure 8c. A Cartesian grid was initially constructed for the far field grid and then split into an unstructured body-fitted grid near the body surface. The grid element is mainly hexahedral. The element size inside the ship bow refinement was controlled to be around 0.01 m and around 1:1:1 in the *X*, *Y* and *Z* directions. In the present work, the *X* direction points downstream with the origin (0, 0, 0) at the cross point of the ship FP and the undisturbed free surface. The *Y* direction points to the ship’s portside. The *Z* direction points upward. The vertical grid size near the free surface was around 0.01 m as well. The boundary layer grid was built for three layers, with the first layer thickness around 0.01 m to control *y*^+^ ~ 150 to remain in the logarithmic layer range (*y*^+^ = 30–200). The *y*^+^ is the non-dimensional distance between the wall and the control point of the first layer grid. Thus, near-wall treatment was required (next discussed for Table 1).

The boundary conditions listed in Table 1 are imposed on the boundary faces shown in Figure 8a,b. *U* is the flow velocity, which is a vector value; *P* is the fluid static pressure (excluding hydrostatic pressure); *ω* is the turbulence dissipation rate; *v_t_* is the turbulence viscosity coefficient; and *k* is the turbulence kinetic energy. *α* is the volume fraction of VOF to distinguish the air–water ratio inside one grid cell. Here, *α* = 0 for air and *α* = 1 for water, so *α* = 0.5 corresponds to the location of the free surface. On the inlet, side and bottom boundaries, the fixed values of uniform inflow were applied as in Equation (1) below:

*U* = (1.248, 0, 0) m/s, *ω* = 2 s^−1^, *v_t_* = 0.0000005 m^2^/s, *k* = 0.00015 m^2^/s^2^, *α* = 0~1. (1)

The *ω*, *μ_t_* and *k* values in Equation (1) were estimated based on free stream turbulence.

As zero gradient condition was specified, the flow variables *Q*, such as *P* (inlet, side and bottom), *μ_t_* (top and outlet), *k* and *α* (hull and sonar dome surface), are substituted into the following Equation (2):

(2) ∇Q=0.

The symmetric condition was used on the mid-plane as shown in Table 1 and Equation (3). It imposes a zero-gradient for all flow variables in the normal direction (*n*) of the plane.

(3) $$\nabla_{n}Q=0.$$

Equation (4) is the so-called pressureInletOutletVelocity condition in OpenFOAM, which was used on the top boundary to ensure no fluid reversely flowing back into the domain. An additional statement: zero tangential velocity (*U_t_*) is forced once inverse flow occurs and was attached along the zero-gradient velocity condition, i.e., *Q = U* for Equation (2).

(4) $$\nabla U=0\;\mathrm{but}\;U_{t}=0\;\mathrm{in}\;\mathrm{case}\;\mathrm{of}\;\mathrm{inverse}\;\mathrm{flow}.$$

On the top boundary, the total pressure was fixed to the reference pressure equal to zero in the infinite far field. However, as Equation (5) states: once the fluid flows into the domain, the dynamic pressure is removed.

(5) $$P=P_{0}=0\;\mathrm{for}\;\mathrm{outflow},\;P=P_{0}-\frac{1}{2}U^{2}\;\mathrm{for}\;\mathrm{inflow}.$$

The boundary condition called inletOutlet was applied as in Equation (6) and describes *ω* and *k* (top and outlet) and *α* (top). It is a variation of the zero-gradient condition which is represented in Equation (2), but if inverse flow occurs, the inflow fixed values in Equation (1) are recovered.

(6) ∇Q=0, but Equation (1) is forced in the case of inverse flow.

Equation (7) is named the outletPhaseMeanVelocity condition in OpenFOAM to ensure the conservation of water and air between flow-in and flow-out. It is a modified zero-gradient velocity boundary condition which was used on the outlet boundary, i.e., *Q = U* for Equation (2), but *U* is adjusted based on mean flux of the two phases in the case of inverse flow.

(7) ∇U=0 but U is adjusted based on the two-phase mean flux in the case of inverse flow.

To maintain the water level from inlet, at outlet a conditional zero-gradient condition variableHeightFlowRate was imposed for *α*, as explained in Equation (8). Accordingly, the *α* solution is secured in the range between 0 and 1.

(8) If α<0, α=0,If 0<α<1, ∇α=0,If α>1, α=1.

No-slip conditions, such as Equation (9,) give zero velocity for the solid surfaces, including the hull and sonar dome.

*U* = (0, 0, 0). (9)

Moreover, near-wall treatment was achieved by applying Equations (10) and (11) for *μ_t_* and *ω*:

(10) $$\mu_{t}=\mu \left(\frac{0.41y^{+}}{\ln\left[\left(1+C_{S}k_{S}^{+}\right)y^{+}\right]}-1\right),$$

wherein the surface roughness was considered in Equation (10) by setting the equivalent roughness height *K_s_* = 100^−6^ and the roughness constant *C_s_* = 0.5. Both are common values.

(11) $$\omega =\sqrt{{\left[\frac{6}{0.075{\left(y^{+}\right)}^{2}}\right]}^{2}+{\left[\frac{k^{+}}{0.41y^{+}}\right]}^{2}},$$

wherein *k^+^* is the non-dimensional turbulence kinetic energy.

Before designing the sonar dome, we needed to make sure our CFD method was reliable. The VV (verification and validation) analysis was conducted for the ship hull with the original sonar dome, and the sonar dome design followed the same CFD setup and method. The VV theory is based on ITTC 7.5-03-01-01 [11]. For verification, at least three different grid densities were suggested to check the grid independence, i.e., grid convergence. For validation, the factor of the safety method with correction factor was proposed to evaluate grid uncertainty (*U_G_*). Here, the refinement ratio between two different grid densities was $\sqrt{2}$ in the *X*, *Y*, *Z* directions which were set for the boundaries initially. The unstructured grid solver constructed the coarse, medium and fine grids as shown in Figure 8d, and their total grid numbers are listed in Table 2. The grid number ratio was controlled around $\sqrt{8}=\sqrt{2}\times \sqrt{2}\times \sqrt{2}$. For validation, the numerical uncertainty is compared with the simulation error. Section 4.1 proves that the CFD method was verified and validated. Accordingly, the medium grid is chosen for the sonar dome design in the consideration of the computational time consumption and a grid size small enough to capture flow field characteristics. For all different geometries, the total grid number was controlled near 1.44 M, with a change of less than 120 grid points. The same verification method was applied to the optimal designs to ensure grid convergence, and their total grid numbers are listed in Table 2.

## 4. Results

In this section, the confidence of our CFD method is examined through VV analysis for a ship hull with the original sonar dome, and then the medium grid is selected for the further sonar dome design. In addition to the resistance values, the detailed flow field around the sonar domes is analyzed to understand the mechanism of resistance reduction and to observe the flow field phenomena.

### 4.1. Verification and Validation for Original Sonar Dome

In Table 3, each value below, *S*_1_, *S*_2_ and *S*_3,_ represents the total ship resistance *R*(N) for fine, medium and coarse grids, respectively. To check the grid convergence, a ratio, *RG*, is suggested:

(12) $$RG=\frac{S_{2}-S_{1}}{S_{3}-S_{2}}.$$

Once *RG* is less than 1, it means the resistance difference between the medium and fine grids is smaller than the difference between the medium and coarse grids. The so-called monotonic convergence is achieved: as the grid number increases, the resistance difference decreases between the two grid densities. Therefore, our CFD method is verified, and grid independence is confirmed.

For validation, the grid uncertainty *U_G_* is estimated as shown in Table 3 and also compared with the error *E%D*, defined as:

(13) $$E\%D=\frac{D-S_{i}}{D},\;i=1,\;2,\;3.$$

wherein *D* is the experimental resistance value. If |*U_G_*| is larger than the |*E%D*| of *S*_1_, the validation is satisfied, i.e., the uncertainty level between the CFD and the experimental value is below the CFD value itself. As shown in Table 3, our CFD method is also validated. In addition, average *y*^+^ = 155, 159 and 162 for *S*_1_, *S*_2_ and *S*_3_, respectively. The *y*^+^ around 150 within 30–200 is confirmed.

As grid density rises, the absolute error is reduced from over-prediction, becoming slight under-prediction. Overall, the errors are considerably small. All errors are less than 4%. The minimal error is less than 1%. To compromise the computational time consumption and the flow field resolution, the *S*_2_ grid, i.e., the medium grid with grid number 1.44 M, was selected for the sonar dome design. Additionally, the *S*_2_ error is merely as small as the over-predicted 1%.

### 4.2. Resistance Reduction for the Sonar Dome Design

Table 4 shows the resistance result of all Type A and B geometries and their resistance reduction *R_d_*, which is calculated as below:

(14) $$R_{d}=\frac{R-S_{2}}{S_{2}}.$$

For both types, with increasing *θ* from 14.967°, the total resistance shows the decreasing trend, i.e., *R_d_* increases but reaches the limit. The resistance reduction effect of Type B is more obvious than Type A’s, e.g., for the same *θ*, Type B’s *R_d_* is larger than Type A’s. It is because Type B allows larger geometry changes, as mentioned in Section 2, to find better solutions. In conclusion, A17.830 is the optimal design for Type A, obtaining nearly 2% resistance reduction. For Type B, the best resistance reduction can reach more than 2%, which is B17.018. With further front-edge fairing on B17.018, B17.018v could reduce resistance by slightly more than 3%. Decreasing *θ* from 14.967° increases the resistance for Type B and only slightly decreases Type A’s resistance but eventually increases the resistance of Type A also. The result of A14.967 (=B14.967) also points out that only changing the dome back slope on the original shape can provide a certain *R_d_* of around 0.8%.

**Figure 9:**
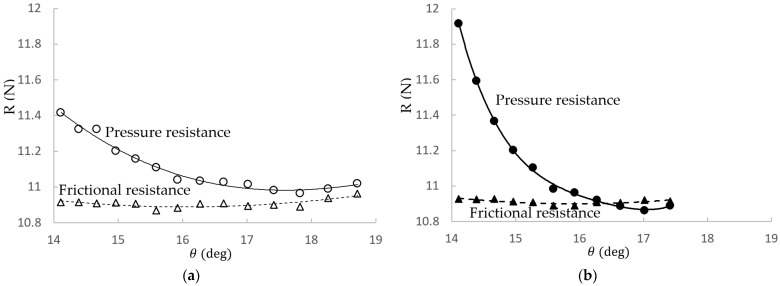
Trend of resistance components. (**a**) Type A and (**b**) Type B.

According to the resistance trend shown in Figure 9, we can conclude that the resistance reduction is mainly due to the decrease in pressure resistance. The next section’s analysis reveals the relation to the smaller flow separation. Type A reduces the pressure resistance to near 11 N, while Type B reduces the value to lower than 11 N, with a greater reduction rate (see the curve slope). The friction resistance is around 10.9 N along *θ* and barely changes because the surface area changes among the different sonar domes are very small. Table 5 lists the resistance components and their reduction quantities. The friction resistance change (reduction, *R_d_*) is very small due to the sonar dome design. The absolute difference |*R_d_*| is less than 0.6%. The friction resistance (*R_F_*) of A17.830 is nearly the same as the original one. The *R_F_* of B17.018 is even slightly increased but after improving becomes B17.018v, and *R_F_* is reduced slightly. The pressure resistance reduction for A17.830, B17.018 and B17.018v is around 4, 5 and 6%, respectively, and it increases as concluded previously. It is obviously larger than the friction resistance reduction.

Grid independence is proven in Table 6, with all *RG* < 1 for those optimal designs. Since the experimental data are not available, the uncertainty *U_G_* is the percentage of the fine grid result *S*_1_, i.e., those *S*_1_ values are assumed to be close enough to the experimental value of the original sonar dome (refer *E%D* = 0.33% in Table 3). All *U_G_* > 0.33%, as seen in Table 6, so the results can be considered validated.

### 4.3. Flow Field Analysis

#### 4.3.1. Axial Velocity Distribution around the Ship Hull

**Figure 10:**
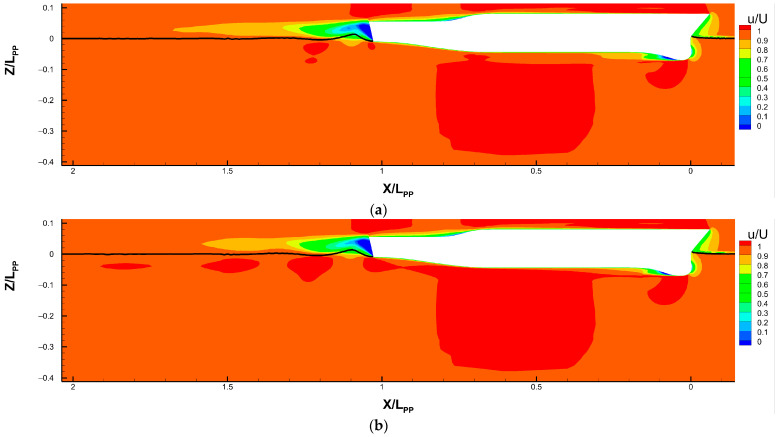

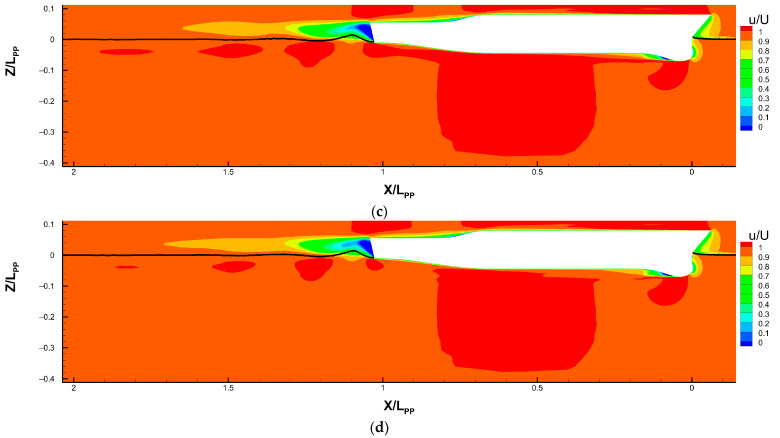
Axial velocity distribution around the ship hull (on *Y* = 0 plane). (**a**) Original sonar dome, (**b**) A17.830, (**c**) B17.018 and (**d**) B17.018v. The black line represents water lines.

Figure 10 provides the global view of the axial velocity distribution around the ship hull with different sonar dome configurations, including the original geometry in Figure 10a, A17.830 in Figure 10b, B17.018 in Figure 10c and B17.018v in Figure 10d. The geometry and domain size are non-dimensionalized by the ship length, *L_PP_*. The axial velocity is the non-dimensional flow velocity *u/U* in the *X* direction, and *U* is the ship speed, 1.248 m/s. Figure 10 plots *u/U* on the mid-plane, i.e., *y =* 0 plane. The *u/U* ranges 0–1 are represented by 11 flooded colors with 0.1 *u/U* increments.

As the inflow reaches the ship bow, the *u/U* drops from 1 to 0 rapidly and forms a stagnation point, i.e., *u/U* = 0, at the sonar dome’s front edge. Going downstream, the boundary layer develops along the solid surface. In particular, behind the back slope of the sonar domes the obvious flow separation occurs. It can be observed in the dark blue area indicating negative *u/U* and reverse flow. In the figures, the separation extends downstream and becomes the sonar dome’s wake. Using *u/U* = 0.8~0.9 as an indicator, the brown (dark yellow) region behind the sonar dome is much thinner and shorter for A17.830 and B17.018 compared to the original. However, for B17.018v, one is slightly larger than the A17.830 and B17.018 ones but still smaller than the original. This is one of proofs that the resistance is reduced successfully by the sonar dome design. The quantity details are discussed in the next section, Section 4.3.2.

Outside the boundary layer, as the flow passes the curved surface, the outer *u/U* is accelerated more than 1, resulting in the dark red region beneath the sonar dome and a large region of *u/U* > 1 along the whole ship bottom. For the original sonar dome, the *u/U* > 1 area is truncated between the sonar dome and the ship bottom. The ship bottom one is separated by the sonar dome’s wake and is located deeper, away from the ship bottom. As the outcome of the sonar dome resistance reduction, A17.830 and B17.018 have the continuous and large *u/U* > 1 area below and close to their sonar dome and ship bottom. They even extend to the ship stern and downstream to form several *u/U* > 1 fragments inside the ship’s wake; in contrast, the *u/U* > 1 area is relatively small for the original. For B17.018v, its *u/U* > 1 area is not as continuous and large as A17.830 and B17.018, but it is still much better than the original one. Higher ship wake velocity implies less momentum loss due to the thinner boundary layer and reflects smaller ship total resistance.

#### 4.3.2. Flow Separation behind the Sonar Dome

**Figure 11:**
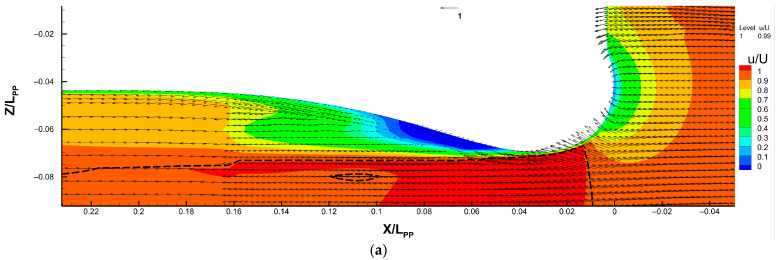

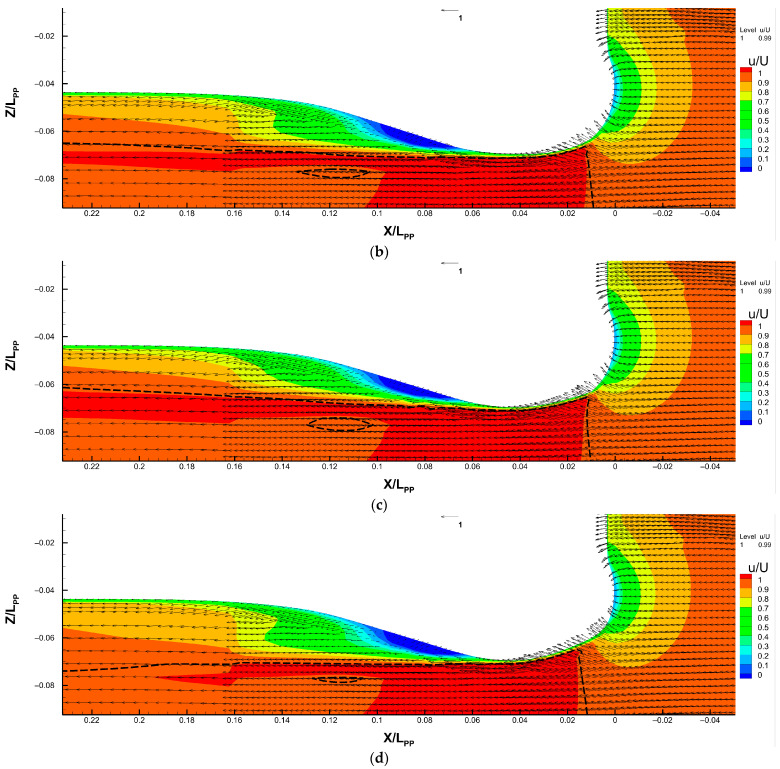
Velocity flow field around the sonar dome (on *Y* = 0 plane). (**a**) Original sonar dome, (**b**) A17.830, (**c**) B17.018 and (**d**) B17.018v. The arrows illustrate the velocity vector field (*u/U*, *v/U*) with the reference vector length for the velocity magnitude equal to 1 showed on the middle top of each figure. The dashed lines represent the *u/U* = 0.99 contour line, i.e., boundary layer location.

Figure 11 shows the local detailed flow field, including the axial velocity magnitude *u/U* and vector field (*u/U*, *v/U*) for the original geometry in Figure 11a, A17.830 in Figure 11b, B17.018 in Figure 11c and B17.018v in Figure 11d. The flow field improvement, i.e., smaller flow separation behind the sonar dome, can be observed clearly for A17.830, B17.018 and B17.018v. Their dark blue area (*u/U* < 0) is visibly smaller than the original one. Inside the dark blue area, the direction of vectors also shows the reverse flow.

**Figure 12:**
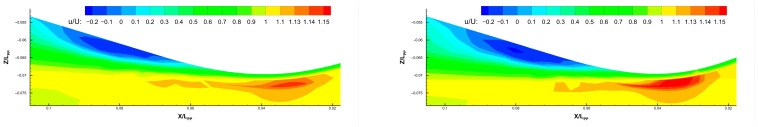
Lowest and highest axial velocity around sonar dome (**left**: B17.018; **right**: B17.018v).

The boundary layer thickness and sonar dome wake length can also be measured from the figures and are listed in Table 7 to support the conclusion regarding the flow field improvement. Based on the definition of boundary layer thickness, the intersection of the contour line of *u/U* = 0.99 (the dashed lines in Figure 11) at the vertical axis is used as a comparison reference. For the original sonar dome, the intersection is at *z/Lpp* = −0.079. It is at *z/Lpp* = −0.065 for A17.830 but at *z/Lpp* = −0.061 for B17.018. The wake length is represented by the horizontal location of the bulge tip of the *u/U* = 0.7 contour line behind the sonar dome, which is the juncture between the yellow- and green-colored areas in Figure 12. It is at *x/Lpp* = 0.155 for the original one and 0.144 for A17.830. There is no clear bulge part among the contour lines for B17.018, e.g., a smooth contour line appears for *u/U* = 0.7. Thinner boundary layer and shorter wake indicate better flow field improvement. Thus, B17.018 is the best, and then A17.830 is better than the original sonar dome.

More resistance reduction basically reflects better flow field performance, except for B17.018v, which shows *z/Lpp* = −0.074 and *x/Lpp* = 0.147 in the abovementioned analysis for Figure 11 and Table 7. Although B17.018v is the further improved shape based on B17.018, and B17.018v indeed provides a lower resistance value, B17.018 has a thinner boundary layer and shorter wake than B17.018v. Outside the boundary layer, the length of the high-speed flow region (*u/U* > 1) of B17.018v is also shorter than those of A17.830 and B17.018, and it is only slightly longer than the original sonar dome. According to Table 7, it is *x/Lpp* = 0.195 for B17.018v, and 0.178 for the original one. It is even longer than *x/Lpp* = 0.232 (beyond the horizontal range of Figure 11) for both A17.830 and B17.018. In fact, as discussed in Section 4.3.1 for Figure 10b,c, their length extends continuously along the ship bottom through the whole ship length to the ships’ sterns.

To explain the resistance reduction of B17.018v, Figure 12 compares the axial velocity *u/U* distribution around the sonar dome, ranging from the lowest and highest value for B17.018 and B17.018v. We can observe that the flow is accelerated higher than *u/U* = 1.15 outside the boundary layer below the sonar dome for B17.018v, but for B17.018 it only can reach *u/U* = 1.14–1.15. The area of *u/U* > 1.15 of B17.018v is quite large as well, which almost equals the area of *u/U* = 1.13–1.15 of B17.018. It proves that the front-edge fairing of B17.018v effectively increases the flow acceleration around the sonar dome. B17.018 and B17.018v have similar flow separation areas behind the dome back slope. Inside the separation area, the reverse flow velocity for B17.018v is lower than *u/U* = −0.2, but it is in a very small region. This is the reason why B17.018v has a thicker boundary layer, longer wake and slightly worse separation, but the resistance can still be reduced. It is because of the much higher flow acceleration below (around) the sonar dome caused by the front-edge fairing. Moreover, it implies that the further optimization of the back slope *θ* for B17.018v is required. In other words, once the sonar dome front edge is faired, *θ* = 17.018 might deviate from the optimal situation.

#### 4.3.3. Surface Pressure Distribution on the Ship Bow and Sonar Dome

**Figure 13:**
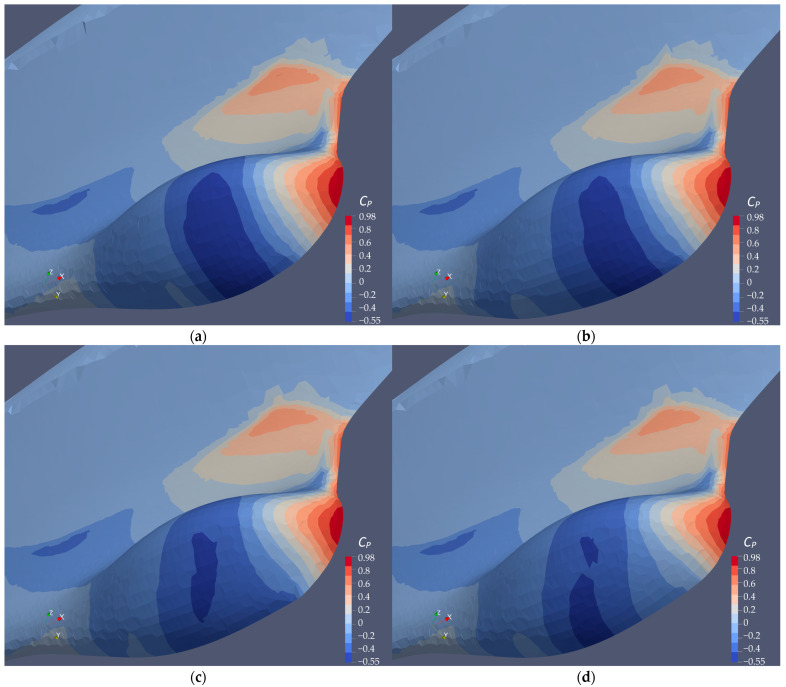
Distribution of pressure coefficients (*C_P_*) on bow surface. (**a**) Original Sonar dome, (**b**) A17.830, (**c**) B17.018 and (**d**) B17.018v.

The comparison of the pressure distribution on the ship hull surface in Figure 13 supports the conclusion regarding the pressure resistance reduction in Section 4.3.1 and the observation of the smaller flow separation in Section 4.3.2 for those improved sonar dome geometries. The pressure distribution in the figure is presented by pressure coefficient *C_P_*, which is calculated as indicated in Equation (15):

(15) $$C_{P}=\frac{P}{0.5\rho U^{2}},$$

where *P* is static pressure (Pa). The *C_P_* range is scaled to the maximal and minimal values among the geometries. Maximal and minimal *C_P_* and the differences between them (Δ*C_P_*) for the different geometries are listed in Table 8.

**Figure 14:**
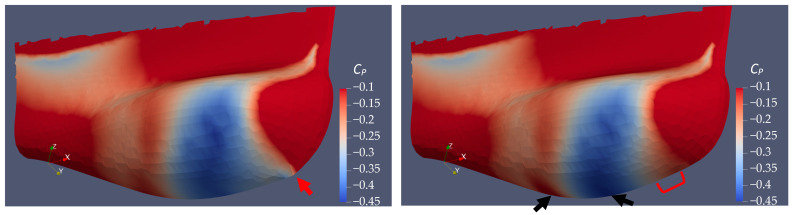
Low pressure distribution (−*C_P_*) on sonar dome surface (**left**: B17.018; **right**: B17.018v). The red arrow points to the sharp tip of the front edge of B17.018 sonar dome. The red bracket shows the front edge fairing for B17.018v. The black arrows indicate lower *C_P_* on the dome bottom and higher *C_P_* on dome slope for B17.018v compared with B17.018.

Since the flow separation area behind the sonar dome back slope is observed to be smaller for A17.830, B17.018 and B17.018v than the original sonar dome (Section 4.3.2, Figure 11), their minimal *C_P_* are all considerably higher than the original one in Figure 13 and Table 8. With their highest *C_P_* all near 1, it means they have less pressure difference between the front and back surface of the dome. Considering the geometry changes are very subtle among the geometries, their surface area, *A*, could be assumed to be the same. The pressure coefficient difference, i.e., Δ*C_P_* in Table 8, could be the direct indicator to evaluate the pressure resistance. For A17.830, the geometry change is limited to the back slope and the dome front edge is not modified. As a result, its maximal *C_P_* = 0.975 near the dome tip is nearly the same as the original one. However, its minimal *C_P_* = −0.49 is still sufficiently lower than the original one (*C_P_* = −0.55). Thus, the *C_P_* distribution of the original and that of A17.830 share the similar pattern seen in Figure 14, but the negative *C_P_* region shows a greater difference. The dark blue area is smaller for A17.830, and the *C_P_* on the back slope is higher, i.e., the lighter blue color is larger and closer to *C_P_* = 0. Δ*C_P_* = 1.46 for A17.830 is certainly smaller than original one, which is Δ*C_P_* = 1.53. The conclusion regarding the pressure resistance reduction is thereby agreed here.

For B17.018 and B17.018v, the pressure distribution is improved by the larger deformation for the dome bottom near front edge. Compared with the original one and A17.830, their negative *C_P_* regions show different patterns with much smaller dark blue areas. Correspondingly, the magnitudes of their maximal and minimal *C_P_* are lower: the maximal *C_P_* decreases to 0.97, and the minimal *C_P_* increases to −0.45. Thus, a better pressure resistance reduction is guaranteed. Their Δ*C_P_ =* 1.42 is significantly smaller than the original one (Δ*C_P_* = 1.53), and smaller than A17.830 as well (Δ*C_P_* = 1.46). To investigate and understand the difference between B17.018 and B17.018v, the *C_P_* distribution plotted in Figure 14 is limited between −0.1 and −0.45 in order to reveal what Figure 13 cannot show.

Comparing B17.018 and B17.018v in Figure 14, e.g., the change gradient between *C_P_* = −0.3 to −0.1 around the sharp tip of the front edge of B17.018 is more sudden. Instead, the *C_P_* change of B17.018v along the front edge is smoother and covers a larger area. Refer to where the red arrow points and compare it with the part that the red bracket indicates. Additionally, the *C_P_* on the back slope of B17.018v is higher. In Figure 14 (right, see the left black arrow), the red color contour area near the slope bottom is darker and larger, i.e., its *C_P_* is closer to −0.1, or even larger than −0.1. As a result, the *C_P_* in the front and back surface of B17.018v are closer, which means a smaller pressure difference. This explains that the pressure resistance reduction is further improved from B17.018 to B17.018v. On the other hand, on the bottom of sonar dome B17.018v, it shows lower pressure distribution, i.e., the dark blue color contour with *C_P_* = −0.45 covers a larger area (see the right arrow in Figure 14 right), which is evidence that the front-edge fairing of B17.018v helps the flow acceleration when fluid passes around the sonar dome outside the boundary layer, as discussed in Section 4.3.2 for Figure 11.

## 5. Discussion

In this paper, firstly a CFD grid system and simulation method using OpenFOAM were verified and validated successfully for the original sonar dome. The medium grid with 1.44M grids was selected for the sonar dome design in order to strike a balance between computational time consumption and flow field resolution. Two types of sonar dome design were proposed. Type A ensured less influence on the interior equipment installation and enabled the re-use of the non-steel dome part. By contrast, Type B allowed larger shape deformation. For Type A, A17.830 achieves almost 2% resistance reduction. Type B could lower the resistance more and the optimal B17.018 provided 2.3% resistance reduction. With proper dome front-edge fairing, B17.018v could even further decrease the resistance reduction, up to 3.3%. The resistance reduction was mainly due to the pressure resistance decrease. The gird dependence and uncertainty level were checked and satisfied as well for A17.830, B17.018 and B17.018v.

Flow field analysis showed smaller flow separation areas, thinner boundary layers and shorter wake lengths behind the sonar dome slope and longer and larger *u/U* > 1 areas around the hull for A17.830 and B17.018 in comparison with the original sonar dome. However, B17.018v resulted in worse than the abovementioned flow field characteristics compared with B17.018. The reason B17.018v could provide better resistance reduction is that the front-edge fairing assisted the flow below the sonar dome accelerate much more with a larger area. The pressure distribution on the bow surface supported the conclusion for the velocity field analysis and pressure resistance reduction. B17.018v showed smoother pressure distribution changes covering a larger area along the front edge than B17.018.

The conclusion addresses the possibility to further improve the flow field for B17.018v. With the faired sonar dome front edge, its back slope, *θ*, should be examined again in future work for the optimal situation. The design loop or iteration between front-edge fairing and back slope may be required.

The remaining challenge and future work are the suitability of the optimal geometry for different ship speeds and full-scale ships since the verification and validation (VV) were only satisfied for one ship speed. For different ship speeds, the CFD prediction would deviate from the experimental value. Different grid generation methods might be required for different speeds. At low speed, the layer grid on the solid surface needs to be generated carefully to resolve the thicker boundary layer. For higher speed, instead, the grid around the free surface should be distributed properly to capture the large ship making wave amplitude. The resistance reduction of the sonar dome design might still be effective at high ship speeds. To consider the scale effect, based on a particular full-scale estimate method, e.g., either the Froude (2D) or Hughes (3D) method, model ship CFD simulation and the full-scale estimate from it should obtain a similar trend for resistance reduction. Performing full-scale CFD simulation will be an option. However, due to the lack of full-scale experimental data, how to perform full-scale simulation or estimate properly will be investigated in the future.

## Figures and Tables

**Table 1:** Boundary conditions.

	*U*	*P*	*v_t_*	*ω*	*k*	*α*
Inlet, side, bottom	Equation (1)	Equation (2)	Equation (1)	Equation (1)	Equation (1)	Equation (1)
Mid-plane	Equation (3)	Equation (3)	Equation (3)	Equation (3)	Equation (3)	Equation (3)
Top	Equation (4)	Equation (5)	Equation (2)	Equation (6)	Equation (6)	Equation (6)
Outlet	Equation (7)	Equation (2)	Equation (2)	Equation (6)	Equation (6)	Equation (8)
Hull, sonar dome	Equation (9)	Equation (2)	Equation (10)	Equation (11)	Equation (2)	Equation (2)

**Table 2:** Grid numbers for different grid sizes.

	Fine	Medium	Coarse
Original	4,258,466	1,441,917	457,872
A17.830	4,258,313	1,441,898	457,845
B17.018	4,258,401	1,441,896	457,838
B17.018v	4,258,393	1,441,868	457,861

**Table 3:** VV result for the ship total resistance *R* with the original sonar dome.

	*S*_1_	*S*_2_	*S*_3_	*D*	*ε*_21_	*ε*_32_	*RG*	*U_G_%D*
*R*(N)	21.937	22.289	22.829	22.01	0.353	0.539	0.653	3.77%
*E%D*	0.33%	−1.27%	−3.72%				Verified	Validated

**Table 4:** Resistance results and reduction *R_d_* for Type A and B sonar domes.

Geometry	*R* (N)	*R_d_* (%)		Geometry	*R* (N)	*R_d_* (%)
A14.113	22.330	−0.193		B14.113	22.841	−2.486
A14.387	22.241	0.206		B14.387	22.516	−1.028
A14.671	22.234	0.238		B14.671	22.291	−0.018
A14.967	22.113	0.781	=	B14.967	22.113	0.781
A15.274	22.063	1.005		B15.274	22.013	1.229
A15.596	21.976	1.395		B15.596	21.873	1.858
A15.929	21.926	1.620		B15.929	21.853	1.947
A16.277	21.941	1.552		B16.277	21.828	2.059
A16.640	21.936	1.575		B16.640	21.791	2.226
A17.018	21.909	1.696		B17.018	21.784	2.257
A17.414	21.879	1.831		B17.414	21.803	2.172
A17.830	21.856	1.934		B17.018h	21.779	2.279
A18.264	21.929	1.606		B17.018v	21.550	3.307
A18.718	21.983	1.364				

**Table 5:** Comparison of resistance components for different sonar dome configurations.

	*R_P_* (N)	*R_F_* (N)	*R_d_* (%) for *R_P_*	*R_d_* (%) for *R_F_*
Original (*S*_2_)	11.398	10.891		
A17.830	10.966	10.891	−3.79%	−0.0046%
B17.018	10.863	10.922	−4.70%	0.28%
B17.018v	10.721	10.830	−5.94%	−0.56%

**Table 6:** Verification result for the optimal designs.

	*S*_1_ (N)	*S*_2_ (N)	*S*_3_ (N)	*ε*_21_	*ε*_32_	*RG*	*U_G_%S*_1_
A17.830	21.647	21.856	22.381	0.210	0.525	0.399	1.21%
B17.018	21.518	21.784	22.140	0.266	0.356	0.747	4.57%
B17.018v	21.391	21.550	22.093	0.160	0.543	0.294	0.93%

**Table 7:** Quantitative comparison for the flow field around the sonar dome measured in Figure 11.

	*u/U* = 0.99 Thickness	Wake Length (*u/U =* 0.7)	*u/U* > 1 Length
Original	*z/Lpp* = −0.079	*x/Lpp* = 0.155	*x/Lpp* = 0.178
A17.830	*z/Lpp* = −0.065	*x/Lpp* = 0.144	*x/Lpp* > 0.232
B17.018	*z/Lpp* = −0.061	Smooth contour line	*x/Lpp* > 0.232
B17.018v	*z/Lpp* = −0.074	*x/Lpp* = 0.147	*x/Lpp* = 0.195

**Table 8:** Maximal and minimal *C_P_*, and the difference between them (Δ*C_P_*) on bow surfaces.

	Maximal *C_P_*	Minimal *C_P_*	Δ*C_P_*
Original	0.9753	−0.5505	1.5259
A17.830	0.9748	−0.4895	1.4643
B17.018	0.9743	−0.4460	1.4203
B17.018v	0.9727	−0.4490	1.4217

## Data Availability

Not applicable.
